# Breaking Free from Nicotine Gum Dependence: Two Unique Paths to Recovery with Psychosocial Support

**DOI:** 10.1192/j.eurpsy.2025.960

**Published:** 2025-08-26

**Authors:** J. Kosor Ljoka, I. Rojnić Palavra, R. Krčelić, D. Bodor, V. Grošić, I. Filipčić

**Affiliations:** 1 University Psychiatric Hospital Sveti Ivan, Zagreb; 2 Faculty of Dental Medicine and Health Osijek, Osijek; 3 University of Zagreb School of Medicine, Zagreb, Croatia

## Abstract

**Introduction:**

Nicotine replacement therapy, particularly nicotine gum, is an effective tool for smoking cessation but may lead to dependence in 6-9% of long-term users. This report presents two patients with prolonged nicotine gum dependence, exploring individualized treatment strategies and their outcomes.

**Objectives:**

To evaluate two personalized treatments for nicotine gum dependence: one using transdermal nicotine patches with psychosocial support, and the other using bupropion with psychosocial support.

**Methods:**

Patient A, a 67-year-old retired technician with a history of anxiety, depression, and past alcohol and benzodiazepine dependence (both in remission), began smoking at 16 (20 cigarettes a day) and quit at 35 due to Leriche Syndrome, switching to 20 pieces of 2 mg nicotine gum daily. In a four-week program, he transitioned to nicotine patches with a gradual dose reduction, alongside psychosocial support. Patient B, a 61-year-old social worker with a history of depression, smoked 40 cigarettes daily until quitting at 51 due to COPD, then developed a long-term dependence on 30 pieces of 4 mg gum. Fifteen days before admission, she abruptly discontinued using nicotine gum. She presented to the program seeking support and strategies for managing cravings, and her treatment focused on psychosocial interventions and medication adjustments.

**Results:**

Both patients successfully achieved abstinence and improved functioning. Patient A transitioned from nicotine gum to nicotine patches, starting at 40 mg daily, reducing to 25 mg after six weeks, and then to 15 mg before discontinuation. Throughout the treatment period, the patient successfully maintained abstinence. Psychological assessments showed stable mood and satisfactory remission. One week into the treatment, patient B reported difficulties in maintaining abstinence after abruptly stopping nicotine gum during a COVID-19 infection. She began experiencing increased depressive symptoms and nervousness, leading to an increase in bupropion (which she used prior to enrolling in the program) dose from 150 mg to 300 mg daily. Active participation in group therapy helped her manage cravings and control anxiety, preventing relapse.

**Conclusions:**

Two distinct approaches, one using nicotine patches and the other pharmacological support, both highlighted the importance of psychosocial support in achieving nicotine gum dependence cessation. Nicotine patches, with their slower absorption, are less rewarding and reduce long-term dependence risk. Despite patient B’s initial struggles after abrupt cessation, the combination of increased bupropion and active therapy participation helped manage cravings and anxiety. Both cases highlight the importance of individualized care and comprehensive psychosocial interventions for sustained nicotine abstinence.

**Disclosure of Interest:**

None Declared

